# On the Causes of Acute Suppurations in the Light of Present Scientific Views

**Published:** 1888-03

**Authors:** A. Luckerman

**Affiliations:** the Laboratory of Surgical Pathology of Kasan.


ARTICLE V.

### ON THE CAUSES OF ACUTE SUPPURATIONS IN THE LIGHT OF PRESENT SCIENTIFIC VIEWS.

---

**(From a dissertation of A. Luckerman, of the Laboratory of Surgical Pathology of Kasan.)**

After stating the literature of the subject, the author details a great number of his own experiments, by which he reaches the following conclusions:

1. Neither chronic, nor mechanical, nor thermic agents of irritation have the power of producing suppuration in the absence of microbes.

2. If suppuration is produced under the influence of the above named irritants, pyogenous fungi were, of necessity, in simultaneous co-operation.

3. Even substances *chemically pure* may be *impure* mycotically, as disinfectants like turpentine, tar, etc.

4. At present, the following fungi are to be considered *causes of suppuration: staphylococcus pyogenes aureus, albus et citreus, streptococcus pyogenes,* and possibly, in fetid abscesses, *bacillus pyogenes fetidus.*

5. Inoculations with staphylococcus and streptococcus when in very large quantities, will usually cause death, and in all cases will produce suppuration.

6. It is to be supposed that said microbes are present in nature very extensively, on account of the frequency of suppurations.

7. They do not seem to be present in air very numerously, nestling so much the more in most of our domestic objects.

8. These microbes may penetrate into the body either by respiration, or by the channels of digestion, or by the skin. The latter channel seems to be the more frequent one. Besides, they may make their invasions by various cutaneous channels, and even, the cutis being intact, through the glandular orifices, as has been demonstrated by Garre's, as well as the author's experiments.

9, As far as the clinical material, accumulated until now, vouchsafes a conclusion, staphylococcus pyogenes shows the more frequent occurrence, and then streptococcus pyogenes.

10. *Rosenbach's* opinion that streptococcus pyogenes was the cause of diffuse phlegmons, and staphylococcus pyogenes the cause of circumscript suppuration, has not been confirmed by other authors.—*Chirurg. Westnik, 1887, Russian.*

